# ADAM12-L confers acquired 5-fluorouracil resistance in breast cancer cells

**DOI:** 10.1038/s41598-017-10468-x

**Published:** 2017-08-29

**Authors:** Xuedong Wang, Yueping Wang, Juan Gu, Daoping Zhou, Zhimin He, Xinhui Wang, Soldano Ferrone

**Affiliations:** 1 Department of Medical Laboratory Science, The Fifth People’s Hospital of Wuxi, The Medical School of Jiangnan University Wuxi, Wuxi, Jiangsu 214005 China; 2 Department of Pathology, The Fifth People’s Hospital of Wuxi, Nanjing Medical University, Wuxi, Jiangsu 214005 China; 3 Department of Biology, College of Arts & Science, Massachusetts University, Boston, MA 02125 USA; 4 Department of Oncology, The Second People’s Hospital of Anhui Province, Anhui Medical University, Hefei, Anhui 230041 China; 5 Cancer Hospital and Cancer Research Institute, Guangzhou Medical University, Guangzhou, Guangdong, 510095 China; 6 Department of Medical Oncology, Massachusetts General Hospital, Harvard Medical School, Boston, MA 02114 USA

## Abstract

5-FU-based combinatory chemotherapeutic regimens have been routinely used for many years for the treatment of breast cancer patients. Recurrence and chemotherapeutic drug resistance are two of the most prominent factors that underpin the high mortality rates associated with most breast cancers (BC). Increasing evidence indicates that overexpression of ADAMs could correlate with cancer progression. However, the role of ADAMs in the chemoresistance of cancer cells has rarely been reported. In this study, we observed that 5-FU induces expression of the ADAM12 isoform ADAM12-L but not ADAM12-S in BC cells and in recurrent BC tissues. The overexpression of ADAM12-L in BC cells following 5-FU treatment results in the acquisition of resistance to 5-FU. ADAM12-L overexoression also resulted in increased levels of p-Akt but not p-ERK. These alterations enhanced BC cell growth and invasive abilities. Conversely, ADAM12 knockdown attenuated the levels of p-Akt and restored 5-FU sensitivity in 5-FU-resistant BC cells. ADAM12 knockdown also reduced BC cell survival and invasive abilities. These findings suggest that ADAM12-L mediates chemoresistance to 5-FU and 5-FU-induced recurrence of BC by enhancing PI3K/Akt signaling. The results of this study suggest that specific ADAM12-L inhibition could optimize 5-FU-based chemotherapy of BC, thereby preventing BC recurrence in patients.

## Introduction

Breast cancer (BC) is the most common malignancy among women worldwide, with an increasing incidence rate in most countries. Despite recent advances in combination therapies, disease recurrence caused by patient treatment failure remains a major clinical problem. Approximately 6–10% of patients have metastatic disease at the time of diagnosis and around 30% of patients initially diagnosed with early-stage BC will eventually suffer a recurrence^1^. Adjuvant systemic chemotherapy is often prescribed for patients with advanced or recurrent BC, although the first treatment option for BC usually encompasses surgical operation. As shown in several meta-analyses, adjuvant systemic therapies reduce the risk for relapse and death^2, 3^. 5-Fluorouracil (5-FU)-based poly-chemotherapy regimens have long been established for the routine treatment of breast cancer patients in clinical settings^4–6^. Furthermore the integration of taxanes into chemotherapy has improved survival benefits in the adjuvant setting^7^. A significant survival advantage of 5-FU-based chemotherapy has been reported in patients with metastatic cancer as well as in those who have undergone surgery^8, 9^. Although such treatments have resulted in an increased in the survival rate of breast cancer patients, many patients treated with 5-FU-based chemotherapy experience recurrence. Indeed, a study performed by Vulsteke, *et al*. revealed that 15.3% of patients with breast cancer suffered a relapse of the disease following treatment with 5-FU-based chemotherapy^10^. This recurrence is predominantly attributed to the development of chemoresistance during treatment. Chemoresistance to 5-FU-based treatments is a complex and multifaceted problem which involves multiple pathways that facilitate increased 5-FU efflux, evasion of apoptotic pathways, replication checkpoint bypass, increased cell proliferation, and increased DNA damage repair^11–15^. A number of targets have been implicated to have a role in 5-FU resistance during breast cancer including COX-2, Survivin, Bcl-2, 14–3–3σ and the miRNA regulator Dicer^16–20^. However, a better understanding of the molecular mechanisms that underlie chemotherapeutic resistance is required for the development of effective 5-FU-based therapeutic strategies for the treatment of breast cancer.

A disintegrin and metalloproteases (ADAMs) comprise a family of zinc-dependent transmembrane proteins. They are characterized by a multidomain structure comprised of prodomain, metalloprotease, disintegrin, and cysteine-rich domains, as well as a transmembrane and cytoplasmic domain^21^. There is increasing evidence that ADAMs are differentially expressed in malignant tumors and may therefore participate in the pathology of carcinomas. Most notably, ADAM family members such as ADAM-9,-10,-12,-15 and 28 have been shown to be associated with cancer progression and may serve as molecular targets for cancer therapy^22–26^. However, relatively little is known about the role of ADAMs in the modulation of chemosensitivity of cancer cells. For this reason, we attempted to characterize the potential role of ADAMs in chemoresistance regulation. In the present study, we sought to investigate the contribution of ADAMs on 5-FU chemoresistance of breast cancer cells. We investigated 6 ADAM family members as part of this study. It is possible that other ADAMs are involved in this phenomenon. However, the gene expression profile of 5-FU-induced drug-resistant MCF-7/5-FU and SKBR3/5-FU cells showed that only ADAM12 of the ADAM family was significantly different in parental and resistant cells (data not shown). Human ADAM12 exists in two forms that arise from alternate splicing; the prototype membrane-anchored protein (ADAM12-L, including 120 and 90 kDa for latent and active forms) and the shorter secreted type form (ADAM12-S, 68 kDa truncated form). With this in mind, we decided to analyze ADAM12 and several of the ADAM gens that have been shown in previous studies to be related to tumorigenesis^22–26^. Surprisingly, ADAM12 isoform ADAM12-L but not ADAM12-S was shown to confer chemoresistance to 5-FU in breast cancer cells. Thus, we speculate that ADAM12 –L represent a promising therapeutic target for the treatment of drug-resistant cancers.

## Results

### Expression of ADAMs in breast cancer cells after treatment with 5-FU

**Figure 1:**
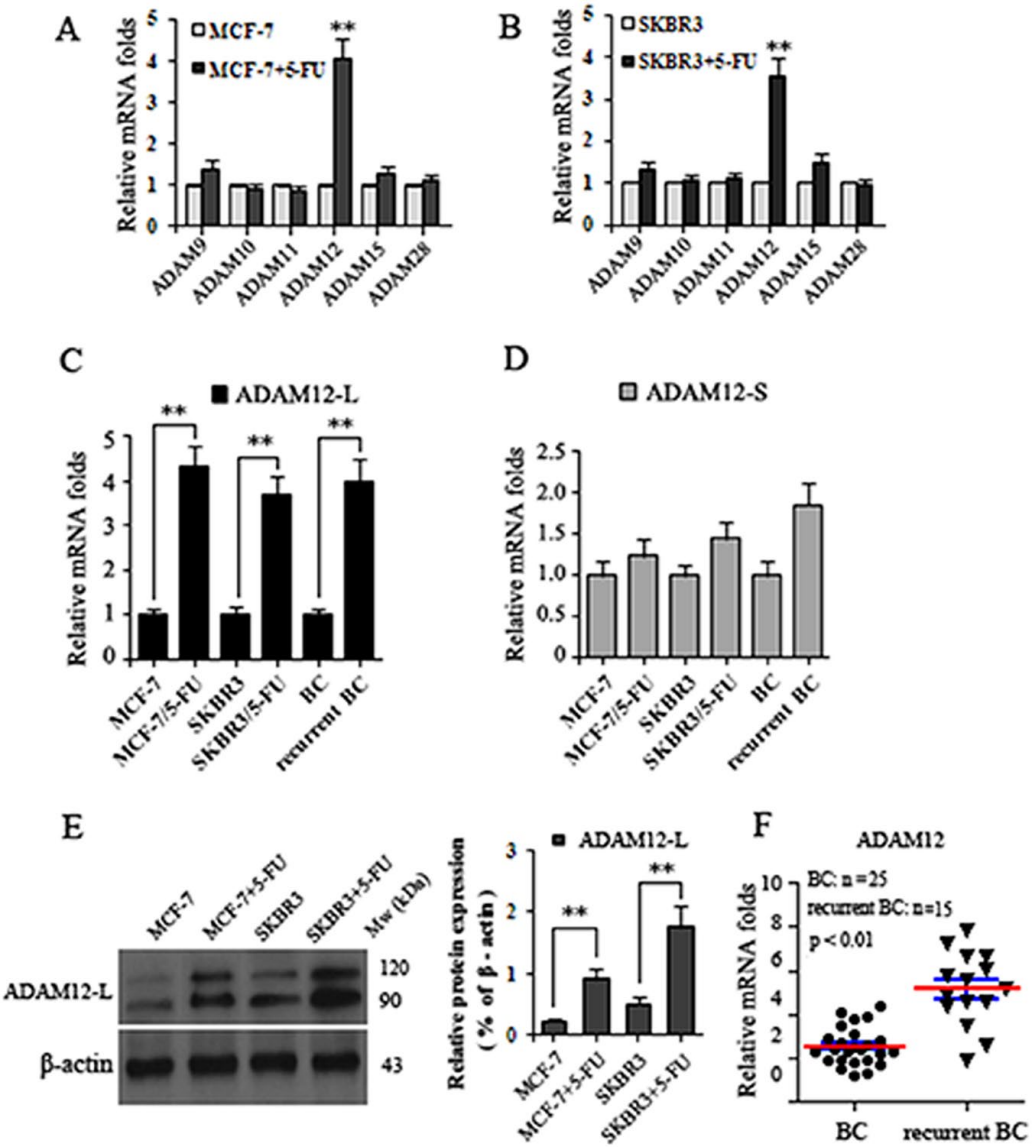
Expression of ADAM family members in breast cancer cells following treatment with 5-FU. **(A)** and **(B)** Relative mRNA levels of ADAM family members determined by qRT-PCR were presented as fold-induction in comparison with untreated control breast cancer cells following treatment with 100 μM 5-FU for 6 days; **(C)** mRNA expression profiles of ADAM12-L isoform determined by qRT-PCR in breast cancer cell lines, primary BC and recurrent BC patient samples are presented as fold-induction following qRT-PCR. **(D)** Relative mRNA levels of ADAM12-S were determined by qRT-PCR in breast cancer cell lines, primary BC and recurrent BC patient samples. **(E)** Representative images of ADAM12-L (including an ~120 kDa latent form, an ~90 kDa active form) protein expression as visualized by Western blotting **(left)**; the relative levels of ADAM12-L protein expression were analyzed from the resultant blots by scanning densitometry **(right)**; **(F)** Relative mRNA levels of ADAM12 in primary BC compared with recurrent BC patient samples. Values for individual patients’ samples are shown. Mean values are represented by a horizontal line; these values were derived from independent runs performed in triplicate and presented relative to lowest expression value observed in the BC patient cohort (=1). All samples for recurrent BC were selected on the basis of documented adjuvant 5-FU therapy. All experiments were carried out in triplicate. Data are shown as means ± SD. *p < 0.05, **p < 0.01.

To investigate the expression of ADAM family members in breast cancer cells after treatment with 5-FU, quantitative real-time PCR (qRT-PCR) was performed to detect ADAM genes in MCF-7 and SKBR3 cells induced following 5-FU treatment for 6 days. Interestingly, the only ADAM gene that was strongly induced by 5-FU (after 6 days) at the mRNA level was ADAM12 (Fig. 1A and B). ADAM12 has two isoforms, ADAM12-L and ADAM12-S. In order to determine which isoform is affected following 5-FU treatment, we analyzed the mRNA expression profiles of both isoforms in breast cancer cell lines using qRT-PCR. We observed a marked increase in ADAM12-L expression compared with ADAM12-S expression following treatment with 5-FU (Fig. 1C and D). ADAM12 protein expression was also examined by Western blotting. Consistent with ADAM12 mRNA expression levels, Western blotting analyses revealed that ADAM12-L protein expression was markedly increased in MCF-7 and SKBR3 cells that were induced following 5-FU treatment for 6 days (Fig. 1E). Interestingly, Several cancer studies have reported that ADAM12 was increased in breast cancer^25–27^; however, little or no information is available for recurrent breast tissues from patients who underwent 5-FU adjuvant therapy. Thus, as part of this analysis, a qRT-PCR was performed in tissues from 25 primary BC and 15 recurrent BC samples following adjuvant therapy with 5-FU (Fig. 1F). We observed that ADAM12 expression was induced by 5-FU, increasing from 2.56 times for primary BC to 5.17 times for recurrent BC. More importantly, we also found a marked increase in ADAM12-L expression compared with ADAM12-S expression in the recurrent breast tissues (Fig. 1C and D). Therefore, we hypothesize that expression of ADAM12-L in BC cells could be mechanistically relevant in relation to the chemoresistance and invasive behavior of recurrent BC cells.

### ADAM12-L facilitates 5-FU resistance in BC cells

**Figure 2:**
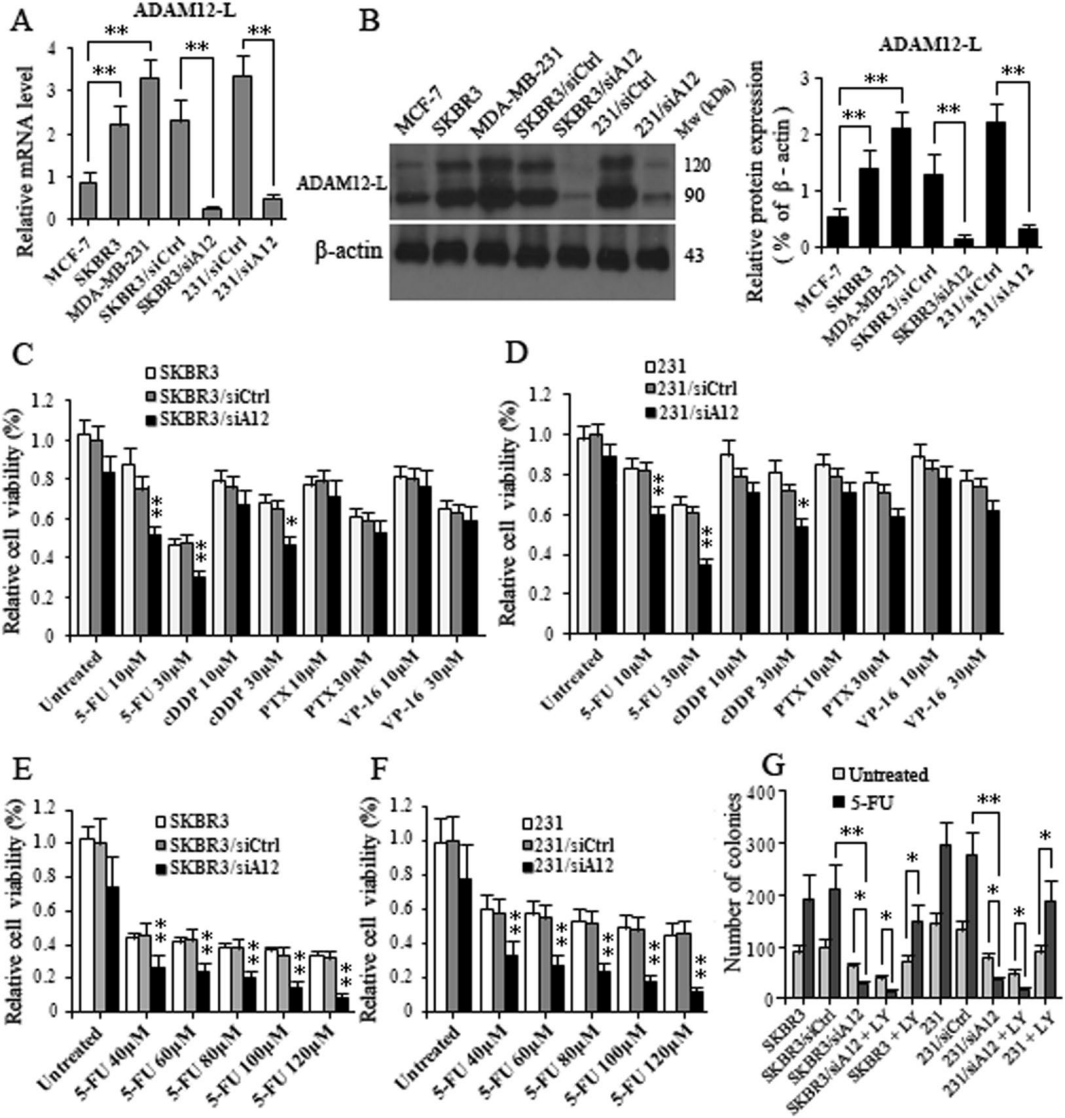
ADAM12-L mediates resistance to 5-FU in breast cancer cells. **(A)** The relative levels of ADAM12-L mRNA were analyzed by qRT-PCR in 3 breast cancer cell lines and SKBR3 and MDA-MB-231 cells following ADAM12 knockdown; **(B)** The representative images of ADAM12-L protein expression are shown as a Western blot **(left)** and the relative levels of ADAM12-L protein expression in 3 breast cancer cell lines and BC cells after ADAM12 knockdown were analyzed from the resultant blots by scanning densitometry **(right)**; **(C–F)** Cell growth curves were generated following CCK-8 assays on SKBR-3 and MDA-MB-231 cells after ADAM12 knockdown or the scrambled and parental cells with increasing concentrations of indicated drugs for 48 h; **(G**) SKBR3 and MDA-MB-231 cells transfected with the indicated siRNA or treated with the Akt inhibitor LY294002 (LY, 20 μM) or after treatment with 5-FU induction (100 μM) were seeded at 500 cells per 6 well plates. Cells were incubated for 14 days at 37 °C to allow colonies to form. Colonies were stained with 2% crystal violet and counted. Each value represents the mean ± SD of three independent experiments (*p < 0.05, **p < 0.01; Student’s t-test).

To assess whether ADAM12-L plays a role in chemoresistance in breast cancer cells, we first analyzed ADAM12-L the expression levels in breast cancer cell lines (MCF-7, SKBR-3 and MDA-MB-231) by qRT-PCR and Western blotting. The data showed that SKBR3 and MDA-MB-231 cell lines expressed high levels of ADAM12-L while the MCF-7 cell line expressed a low level of endogenous ADAM12-L mRNA and protein (Fig. 2A and B). Next, a specific siRNA- targeting human ADAM12 (siA12) lentivirus and non-silencing control siRNA lentivirus, which was used as a scramble control (siCtrl) were generated and used to infect SKBR3 and MDA-MB-231 cells; both of these cell lines harbor high endogenous ADAM12-L levels. The results of qRT-PCR and Western blotting analyses revealed that ADAM12-L mRNA and protein expression levels were effectively suppressed in both ADAM12-siRNA virus-infected SKBR3 and MDA-MB-231 cells compared with the scramble control virus-infected cells. The knockdown ratio was approximately 85.3% and 81.6% in SKBR3 and MDA-MB-231 cells, respectively (Fig. 2A and B).

Cell survival assays (CCK-8) were performed in various kinds of breast cancer cells following treatment with a panel of chemotherapy agents: the DNA topoisomerase II inhibitor (etoposide; VP-16), a microtubule stabilizer (paclitaxel; PTX), a DNA alkylating agent (cisplatin; cDDP) and an anti-metabolite (5-fluorouracil; 5-FU). Interestingly, silencing of ADAM12 in SKBR3 and MDA-MB-231 cells resulted in a statistically relevant increase in sensitivity to 5-FU (10 µM and 30 µM treatments) and cDDP (30 µM treatment) compared to parental cells. Similar increases in sensitivity to PTX and VP-16 were not observed in the ADAD12-silenced cells. These results reveal that ADAM12-silenced SKBR3 and MDA-MB-231 cells were the most sensitive to 5-FU (Fig. 2C and D), suggesting that ADAM12-L might be involved in the resistance of breast cancer cells to 5-FU. In addition, the viabilities of ADAM12-silenced SKBR3 and MDA-MB-231 cells decreased upon treatment with 5-FU in a dose-dependent manner, and the increase in sensitivity to 5-FU following silencing of ADAM12 expression was also dependent of dose (Fig. 2E and F). Moreover, results from the colony formation assay also clearly demonstrated increased sensitivity to 5-FU following silencing of ADAM12. As expected, the colony numbers associated with both ADAM12 knockdown cell lines were significantly less than those of the scramble control cells. In addition, the Akt inhibitor, LY294002 (LY, 20 μM), attenuate the proliferative ability of BC cells that were induced by 5-FU. LY294002 (LY, 20 μM) was even more effective at inhibiting the proliferative ability of ADAM12-silenced cells (Fig. 2G). These data suggest that ADAM12-L inhibition causes sensitization to 5-FU in BC cells. These results most likely reflect an association between ADAM12-L expression, Akt activity and sensitivity to 5-FU.

### ADAM12-L affects 5-FU resistance in BC cells though the PI3K/Akt pathway

**Figure 3:**
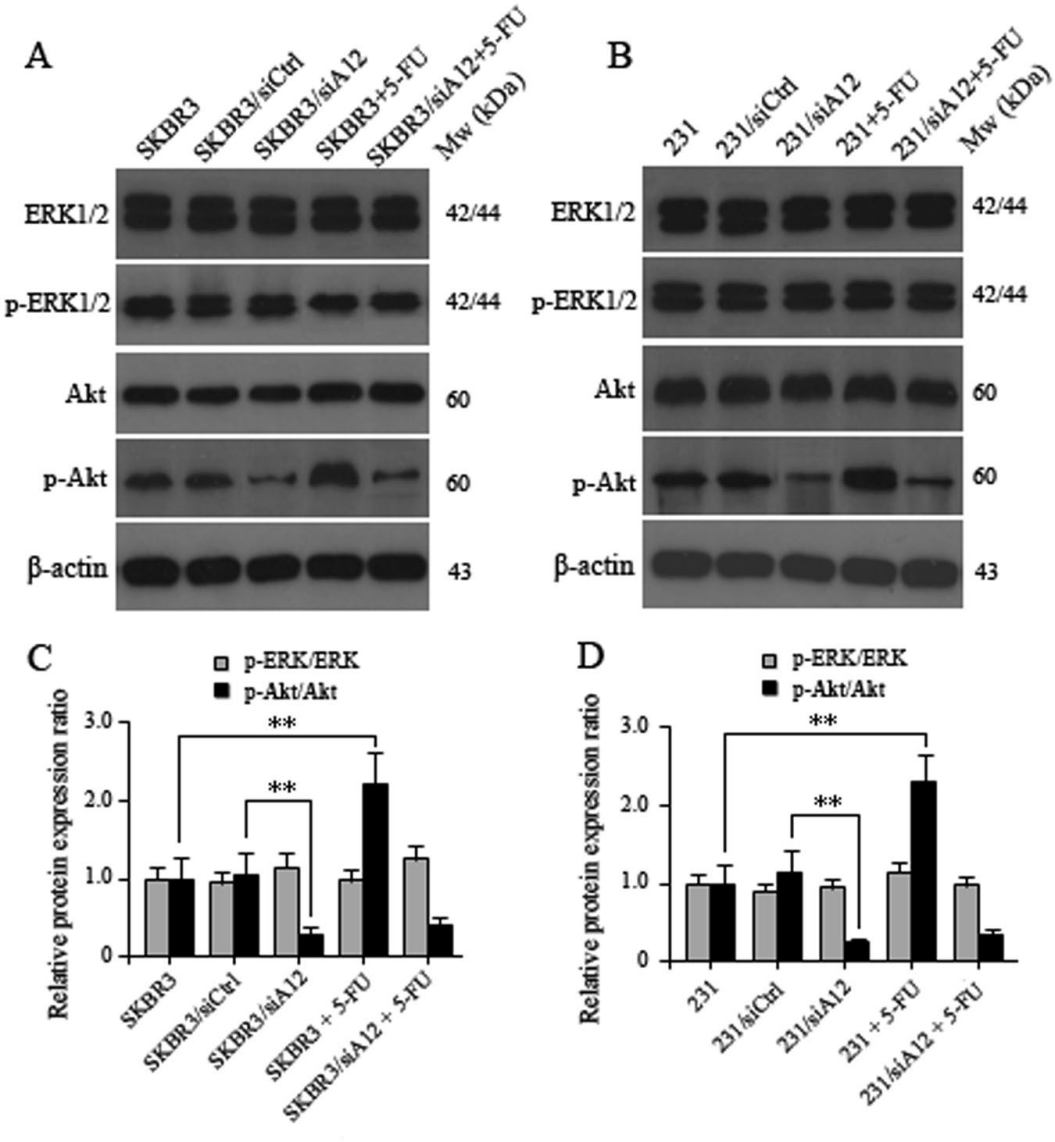
The effects of 5-FU-mediated regulation of ADAM12-L on the expression of PI3K/Akt signaling molecules in breast cancer cells. SKBR3, SKBR3/siA12, MDA-MB-231 and 231/siA12 cells were each treated with 100 μM 5-FU for 6 days, respectively. Cell lysates were subjected to Western blotting analysis. **(A)** Representative images of protein expression of p-ERK1/2 (Tyr202/204) and p-Akt (S473) in SKBR3 and SKBR3/siA12 cells after 5-FU induction compared to the untreated control groups. **(B)** Similar results were observed in MDA-MB-231 and 231/siA12 cells. **(C)** and **(D)** Quantification of the resultant Western blot bands following scanning densitometry: the values are presented relative to β-actin in all cell samples. The values represent the means ± SD of three independent experiments (*p < 0.05, ** < 0.01; Student’s t-test).

To investigate the potential mechanism that underlies ADAM12-L-mediated 5-FU resistance, we investigated the effect of ADAM12 on the downstream activation of the MAPK and PI3K pathways. As part of this analysis, we analyzed both Akt and ERK1/2 to determine whether the latter pathways facilitated ADAM12-L-mediated effects on chemoresistance in breast cancer cells. Western blotting was performed to analyze 5-FU-dependent activation of Akt and ERK1/2. The results showed that the ratios of phosphorylated Akt and total Akt levels (p-Akt/Akt) were significantly decreased in SKBR3 and MDA-MB-231 cells following ADAM12 knockdown compared with the scramble control cells (Fig. 3). Notably, compared with the untreated cells, a significant increase in the ratio of p-Akt/Akt was observed in SKBR3 and MDA-MB-231 cells after 6 days treatment with 100 μM 5-FU. Conversely, compared to their untreated scramble groups, the ratio of p-Akt/Akt did not significantly change following treatment of SKBR3/siA12 and 231/siA12 cells with 5-FU (Fig. 3). Furthermore, in the analyzed cell lines, the ratios of phosphorylated ERK1/2 and total ERK1/2 levels (p-ERK/ERK) were not markedly changed between 5-FU-treated and 5-FU-untreated groups or ADAM12 knockdown and scramble control groups (Fig. 3). These data suggest that ADAM12-L causes chemoresistance to 5-FU via activation of p-Akt and there is a correlation between ADAM12-L expression and Akt activity in breast cancer cells treated with 5-FU.

### ADAM12-L-mediated signaling in BC invasion

**Figure 4:**
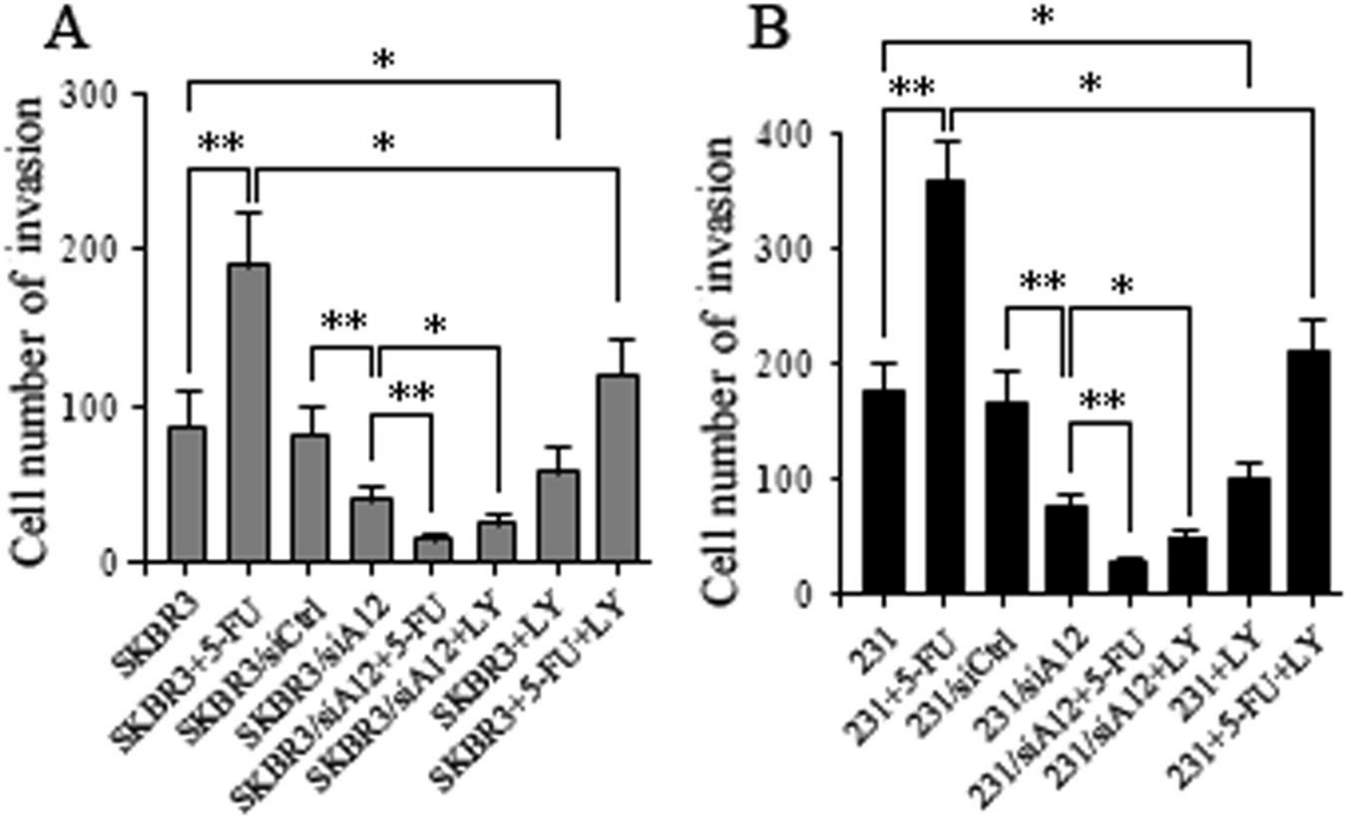
5-FU-mediated regulation of ADAM12-L induced invasion of breast cancer cells. 5-FU induced invasion in breast cancer cells **(A)** SKBR3 and SKBR3/siA12 and **(B)** MDA-MB-231 and 231/siA12. Six days after the addition of 5-FU to breast cancer cells, relative differences in invasiveness were analyzed by Matrigel invasion assays. Compared with control cells, the invasiveness of breast cancer cells following induction with 5-FU was enhanced. The 5-FU-induced invasiveness observed for breast cancer cells was dependent on ADAM12 activity, as demonstrated by the attenuation of 5-FU-induced invasiveness following treatment with the Akt inhibitor, LY294002 (LY, 20 μM). The values represent means ± SD following 3 independent experiments performed in triplicate. Statistical differences were calculated using the Student’s t-test with *p < 0.05, **p < 0.01.

**Figure 5:**
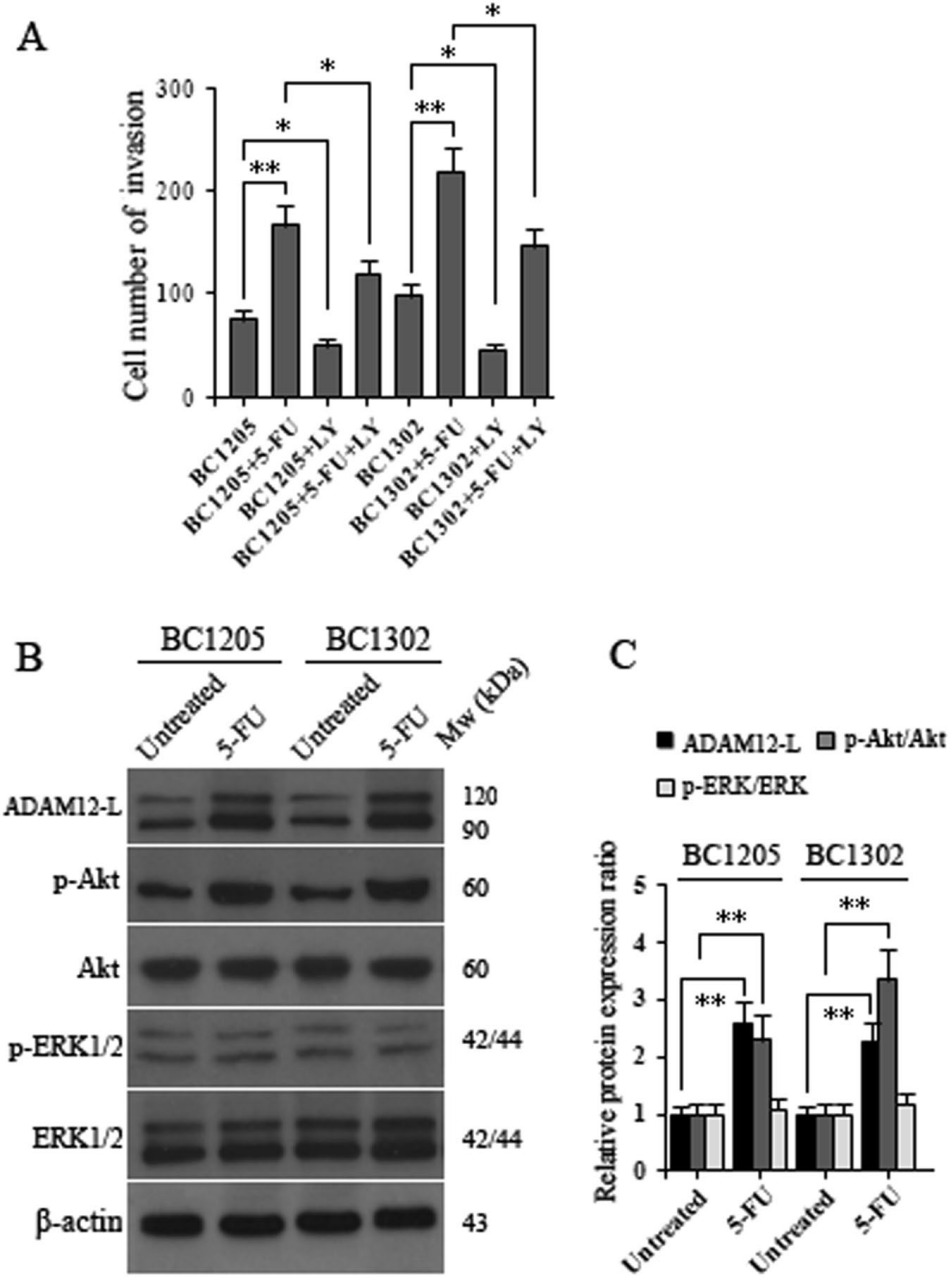
Expression of ADAM12-L and PI3K/Akt in 5-FU-induced primary breast cancer cells and effects of Akt activity on invasiveness. **(A)** 5-FU-induced invasion in primary BC1205 and BC1302 breast cancer cells. Six days after the treatment of BC1205 and BC1302 cells with 5-FU, relative differences in invasiveness were analyzed by Matrigel invasion assays. Compared with untreated cells, the invasion ability of primary breast cancer cells induced by 5-FU was enhanced, as demonstrated by the attenuation of 5-FU-induced invasiveness following treatment with the Akt inhibitor, LY294002 (LY, 20 μM). The values represent means ± SD following 3 independent experiments performed in triplicate. Statistical differences were calculated using the Student’s t-test with *p < 0.05, **p < 0.01. **(B)** Western blot images of ADAM12-L, p-ERK1/2 (Tyr202/204) and p-Akt (S473) in primary breast cancer BC1205 and BC1302 cells in comparison with untreated control groups following induction with 5-FU. **(C)** Quantification of the resultant Western blots was performed by scanning densitometry; the values are presented relative to β-actin in all cell samples. Values represent means ± SD following three independent experiments (*p < 0.05, ** < 0.01; Student’s t-test).

Since ADAM12 has been linked to increased invasiveness of breast cancer cells^26, 27^, we next investigated whether 5-FU-mediated induction of ADAM12-L caused the increased invasion of 5-FU-resistant breast cancer cells. Resistant vital breast cancer cells were treated with 100 μM 5-FU for 6 days and subsequently seeded on Matrigel invasion chambers. The results showed that the number of invasive SKBR3/siA12 and 231/siA12 cells was dramatically decreased compared with the number of invasive SKBR3/siCtrl and 231/siCtrl cells (Fig. 4). Compared with SKBR3, MDA-MB-231, BC1205 and BC1302 cells, significant increases in the number of invaded cells were observed for those cells that were treated with 5-FU treatment (Figs 4, 5A). However, in the presence of LY294002 (LY, 20 μΜ), invasion capacities of breast cancer cells were attenuated to varying degrees (Figs 4, 5A). Moreover, the number of invasive cells in the LY294002-treated SKBR3/siA12 and 231/siA12 groups were dramatically reduced (Fig. 4). These data suggest that there is a correlation between Akt activity and 5-FU-induced invasiveness, and 5-FU-mediated induction of ADAM12-L causes increased invasion of 5-FU resistant breast cancer cells. Moreover, to further analyze the correlation between Akt activity and 5-FU treatment, we observed that the treated cells exhibited a higher p-Akt/Akt ration and increased expression of ADAM12-L (Fig. 5B and C). It is likely that these phenomena play a critical role in the invasiveness of breast cancer cells.

### Therapeutic efficacy of ADAM12 silencing following 5-FU treatment

**Figure 6:**
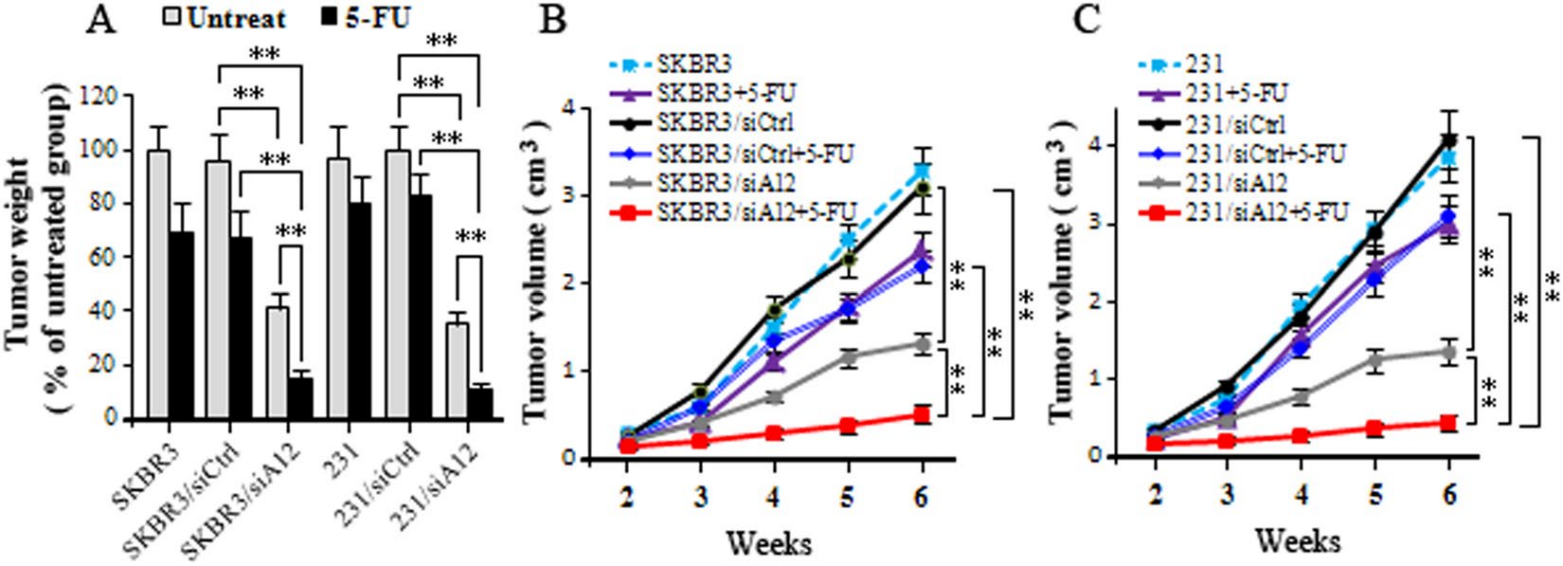
The effects of 5-FU-mediated regulation of ADAM12 on *in vivo* tumorigenicity. **(A)** Tumors produced by MDA-MB-231, 231/siCtrl and 231/siA12 cells (5 × 10^6^) were injected subcutaneously into the mammary glands of nude mice per mouse respectively (n = 4). Upon development of tumors within 9 days, the mice were randomly distributed into two groups; those that were treated by intraperitoneal injection with 5-FU (1.5 mg/kg) and those that were untreated with 5-FU; **(B)** and **(C)** Tumor growth curves were monitored during the experimental period (n = 4). Data represent the means ± SD following three independent experiments. *p < 0.05, **p < 0.01 vs. control.

We next analyzed the effect of ADAM12 silencing using a xenograft tumor growth model and 5-FU treatment. As expected, the tumors formed from SKBR3/siA12 or MDA-MB-231/siA12 cells grew slower than those formed from the scramble control cells. Furthermore, SKBR3/siA12 or MDA-MB-231/siA12 cells treated with 5-FU resulted in an even greater reduction in tumor weight compared with treatment with scramble control and 5-FU or ADAM12 siRNA alone. The tumor growth curves and harvested tumor weights are shown in Fig. 6. These xenograft experiments clearly demonstrate that ADAM12 silencing can greatly improve the therapeutic efficacy of 5-FU in breast cancer.

## Discussion

There is increasing evidence that ADAMs are differentially expressed in malignant tumors and may therefore participate in the pathology of carcinomas. It is interesting to note that some the ADAM family members play an important role not only in tumor growth, invasion and metastasis but also in chemoresistance and recurrence of malignant tumors. Previous studies have shown that ADAM12 is a key enzyme implicated in ectodomain shedding of membrane-anchored heparin-binding epidermal growth factor (EGF)-like growth factor (proHB-EGF)-dependent epidermal growth factor receptor (EGFR) transactivation to activate the EGFR signaling pathway^28, 29^, cleave delta-like 1 to activate the Notch signaling pathway^30^, interact with the type II receptor to activate the TGF-beta signal pathway^31^, interact with β1-integrin to regulate cell migration^32^, and can promote angiogenesis^33^. Recently, ADAM12 was found to be highly expressed in breast cancer patients. As a consequence, the function of ADAM12 in stimulating cell proliferation, invasion and metastasis, and chemoresistance was explored. Some studies have shown that ADAM12 expression levels could be used to predict resistance to chemotherapy in ER-negative breast tumor^34–36^. It should be noted that there are two isoforms of ADAM12, ADAM12-L and ADAM12-S. In this study we observed that the expression of ADAM12-L was significantly elevated in different BC cell lines following treatment with 5-FU. Conversely, ADAM-S expression remained relatively stable following 5-FU treatment. For this reason, we further analyzed ADAM12-L expression profiles in relation to chemoresistance as part of this study. Indeed, recently, it has been reported that ADAM12 was elevated in claudin-low tumor and a part of stromal, mammosphere, and EMT gene signatures, which were all associated with breast tumor-initiating cells (BTICs). Thus, ADAM12 may serve as a novel marker and/or a novel therapeutic target in BTICs^27, 37^. However, the correlation between drug-induced chemoresistance and the expression of potential drug target molecule (along with the related mechanisms) such as ADAM12 has yet to be fully elucidated.

In the present study, we demonstrated for the first time that ADAM12-L plays a crucial role in 5-FU-resistant breast cancer cells. In order to investigate this in more detail, 5-FU inducibility of ADAM family members was determined in BC cell lines, and in primary and recurrent BC tissues. We observed that only ADAM12-L expression was increased in 5-FU-resistant BC cells and recurrent BC tissues upon comparison with 5-FU-sensitive BC cells and primary BC tissues. In addition, our results showed that knockdown of ADAM12 abrogated breast cancer cell proliferation and invasive abilities, inhibited xenograft tumor growth, as well as sensitizing breast cancer cells to 5-FU. Moreover, we determined that the mechanistic basis for ADAM12-mediated tumor growth, invasion and resistance to 5-FU in breast cancer cells, occurs at least in part, via regulation of the PI3K/Akt signaling pathway. Following silencing of ADAM12 expression, SKBR3 and MDA-MB-231 cells became significantly sensitive to 5-FU and slightly sensitive to cDDP, suggesting that ADAM12-L might play a role in the resistance of breast cancer cells to 5-FU. Although the viability of SKBR3 and MDA-MB-231 cells decreased upon treatment with 5-FU in a dose-dependent manner, the increase in sensitivity to 5-FU following ADAM12 silencing was also dose-dependent, suggesting the presence of a specific ADAM12-mediated mechanism of resistance to 5-FU. Mechanistically, ADAM12-L dependent differences were observed in both MAPK and PI3K/Akt signaling pathways. Differences in these pathways could account for differences in cell survival and invasiveness, respectively. We observed that p-Akt levels were increased in SKBR3, MDA-MB-231, and primary breast cancer BC1205 and BC1302 cells following induction with high 5-FU concentrations (100 μM) and longer incubation times (6 d); conversely, phosphor-ERK1/2 levels were not significantly altered. As expected, p-Akt levels were decreased in SKBR3 and MDA-MB-231 cells after knockdown of ADAM12. These *in vitro* assays showed that continuous inhibition of Akt phosphorylation by LY294002 has a substantial effect on proliferation, invasion and chemoresistance in 5-FU-resistant BC cells. Higher p-Akt levels following induction by 5-FU in SKBR3 and MDA-MB-231 cells could account for positive ADAM12-L mediated effects in relation to cell survival. In contrast, reduced p-Akt levels in SKBR3 and MDA-MB-231 cells after knockdown of ADAM12 could be correlated to increased cell death. In addition to cell growth and chemoresistance, the regulation of ADAM12-L by 5-FU might contribute to enhanced invasiveness. Because the treatment of breast cancer cells with LY294002 reduced 5-FU induced invasiveness, p-Akt is able to mediate 5-FU induced invasiveness of breast cancer cells.

In conclusion, we have demonstrated, for the first time, that ADAM12-L is a potential target molecule involved in 5-FU induced chemoresistance and enhanced invasion of breast cancer cells following modulation of the PI3k/Akt pathway. Thus, specific inhibition of ADAM12-L in future therapy regimens could optimize 5-FU-based chemotherapy and prevent the recurrence of breast cancers.

## Material and Methods

### Patients, specimens and preparation of primary BC cells

A total of 25 fresh primary breast cancer and 10 adjacent non-cancerous breast tissues as well as 15 recurrent breast cancer specimens were collected from the Wuxi Clinical Hospital of Nanjing Medical University. The Institutional Review Board of Wuxi Hospital and Nanjing Medical University approved all aspects of this study. Methods were carried out in accordance with approved guidelines and informed consent was obtained from all subjects. Clinicopathological classification and staging were assessed according to the American Joint Committee on Cancer (AJCC) criteria. Follow-up information was obtained following the review of patient medical records. Primary breast cancer BC1205 and BC1302 cells were prepared from pathological grade III BC specimens collected directly after surgery. The sections were treated as follows: tumor tissues were washed in HEPES-buffered saline, homogenized and treated for 30 min with 0.025% trypsin/EDTA solution at 37 °C. The resultant cell homogenate was passed over an 80 mm cell strainer and the cell suspension was centrifuged (200 g, 5 min). After 3 washes with medium (DMEM, 10% FCS), the cells were seeded out for propagation and kept under differentiating conditions.

### Cell culture and transfection

Human breast cancer lines including MCF-7, SKBR-3 and MDA-MB-231 were obtained from American type culture collection (ATCC, Manassas, VA). Cells were cultured in DMEM (Gibco, Los Angeles, CA) supplemented with 10% fetal bovine serum (Invitrogen). SKBR3 or MDA-MB-231 cells were cultured in 6-well plates and lentiviral-based RNA knockdown was used for silencing of ADAM12. The lentivirus stocks were generated using the Lentiviral Packaging Mix using the 293FT cell line according to the manufacturer’s protocol (Invitrogen). The sequence of the siRNA that was used to target ADAM-12 was 5′-GGAAGAGCUGAUGAAGUUGTT-3′ and the sequence of the scramble siRNA was 5′-UUCUCCGAACGUGUCACGUTT-3′ ^38^. siRNAs for target genes were synthesized and modified accordingly (Invitrogen). In a previous study pertaining to breast cancer, ADAM12 was reported as having two alternative spliced forms: a transmembrane form (ADAM12-L) and a secreted form (ADAM12-S)^26^. ADAM12-L and -S share a high overall sequence homology, differing only in the transmembrane domain (absent in ADAM12-S) and a C terminus that is distinct in each isoform. ADM12-L (transcript variant 1, exons 1–18, 20–24) encodes the long, transmembrane protein isoform. ADAM12-S (transcript variant 2, exons 1–19) gives rise to the short, secreted protein isoform. In this study, the ADAM12 siRNA is specific for exon 2 and was therefore designed to target both splice forms of ADAM12.

### Quantitative reverse transcription PCR

Total RNA was extracted from tissues using TRIZOL reagent (Invitrogen, Carlsbad, CA, USA) according to the manufacture’s protocol. An equal amount of RNA (10 μg) was reverse-transcribed into cDNA by Reverse Transcriptase (Invitrogen) according to the manufacturer’s instructions. Real-time quantitative PCR was performed with SYBR Green PCR Master Mix ( × 2) (Applied Biosystems, Carlsbad, CA, USA). The primers for the target genes were as follows: ADAM12: 5′-GCAGTTTCACGGAAACCCAC-3′ and 5′-ACACGTGCTGAGACTGACTG-3′; ADAM12-L: 5′-CAGCCAAGCCTGCACTTAG-3′ and 5′-AGTGAGCCGAGTTGTTCTGG-3′; ADAM12-S: 5′-GCTTTGGAGGAAGCACAGAC-3′ and 5′-TCAGTGAGGCAGTAGACGCA-3′; β-actin: 5′-CATGT ACGTTGCTATCCAGGC-3′ and 5′-CTCCTTAATGTCACGCACGAT-3′. The β-actin locus was used as in internal control.

### Western blot analysis

Total protein from cells was extracted in lysis buffer (Pierce, Rockford, IL) and quantified using the Bradford method. Samples of protein (50 μg) of were separated by SDS-PAGE. Proteins were transferred to polyvinylidene fluoride membranes (Millipore, Billerica, MA, USA) and incubated overnight at 4 °C with antibodies against ADAM12 (1:200; Santa Cruz), AKT (pan-AKT and p-AKT), ERK1/2 (pan-ERK and p-ERK1/2 Tyr202/204) (1:1000; Santa Cruz) and a mouse monoclonal antibody against β-actin (1:2000; Santa Cruz). LY29004 was utilized as an AKT inhibitor (Cell signaling, Waltham, MA). After incubation with peroxidase-coupled IgG at 37 °C for 2 hours (2 h), the immunoreactive proteins were detected using chemiluminescence (Pierce, Rockford, IL). After exposing the blots to X-ray film, quantitation was performed by scanning the films obtained from a minimum of 3 independent experiments using an imaging densitometer (Bio-Rad, Philadelphia, PA).

### Cell viability assay and chemotherapeutic agents

Cells were plated at a density of 2 × 10^3^ cells in 96-well plates. Each well contained 200 μl of complete medium, and 3 replicates were prepared per group. After incubation for 48 hours at 37 °C, 10 μl of cell counting kit-8 (CCK-8) was added into each well and then incubated for 2 h. The absorbance was measured at 450 nm and then referenced at 630 nm using a microplate reader. Etoposide, paclitaxel and cisplatin (10 μM and 30 μM) were independently dissolved in dimethylsulfoxide (DMSO). 5-FU was dissolved in sterile water. All of the chemotherapeutic agents were obtained from Sigma (Sigma-Aldrich, St. Louis, MO).

For the colony formation assay, cells that were exposed to 5-FU and treated with ADAM12-siRNAs or the scramble control were plated onto 6-well plates (600 cells/well). The cells were then cultured at 37 °C and 5% CO_2_ for 2 weeks until visible clones were formed. The medium was changed every three days during this time. The clones were washed three times with PBS and fixed with 4% paraformaldehyde for 15 min. The clones were subsequently stained with 2% crystal violet solution for 15 min. After washing, the number of colonies was counted under a microscope. All experiments were performed in triplicates on three independent occasions.

### Invasion assays

The cell invasion assay was performed using a 24-well Transwell chamber with a pore size of 8 μm (Costar, Cambridge, MA). Each insert was coated with 50 μl of Matrigel (1:3 dilution;BD Bioscience, San Jose, CA). The cell suspensions were prepared in serum-free DMEM (1 × 10^4^ cells in 300 μl medium) and transferred to the upper Matrigel chamber and incubated for 48 h. Medium supplemented with 15% FBS was used as a chemoattractant and added to the lower chamber. After incubation, the non-invading cells on the upper membrane surface were removed with a cotton swab, and the cells that passed through the filter were fixed with 4% paraformaldehyde and stained with the HEMA 3 stain set (Fisher Scientific, Pittsburgh, PA) according to the manufacturer’s instructions. The number of invading cells was calculated under the microscope. All experiments were performed in triplicates and repeated three times.

### Xenograft experiments

The animal experimental protocol was conducted in accordance with the guidelines of the “Animal experiment rules in Nanjing Medical University” established by the Experimental Animal Center of Nanjing Medical University (Nanjing, China), and was approved by the Institutional Animal Care and Use Committee of the Nanjing Medical University. All mice were obtained from the Experimental Animal Center of Nanjing Medical University. Female BALB/c nude mice (4–5 weeks old) were maintained under sterile and controlled environmental conditions (22 °C, 50 ± 10% relative humidity, 12 h light-dark cycle, autoclaved bedding), with food and water given ad libidum. Following 7 days of quarantine, the mice were included in the study. To determine tumor volume, two axes of the tumors were measured using a digital Vernier caliper. Tumor volumes were calculated using the following formula: length × width^2^ × 0.5. In order to analyze of the effect of ADAM12, siRNA-ADAM12, scramble control and parental cells were injected into the mammary glands of the mice, respectively. Tumors were allowed to grow until they reached a size of approximately 50–150 mm^3^ (day 9) at which point the first group received placebo (PBS) and the second group received chemotherapy by intraperitoneal injection (5-FU; 1.5 mg/kg, twice a week); each treatment group contained four animals. The tumors were measured in two dimensions 2–3 times a week using a calliper.

### Statistical analysis

Statistical analysis was performed using SPSS software (Version 17.0 SPSS). All quantitative results were reported as means ± standard deviation (SD) for at least three independent experiments. The data were assessed using the Student’s two-tailed t-test. p < 0.05 was considered statistically significant.
